# One Hundred Cerebral Microhemorrhages: Rethinking Cerebral Amyloid Angiopathy in the Era of Amyloid-Targeted Antibodies

**DOI:** 10.7759/cureus.105106

**Published:** 2026-03-12

**Authors:** Amber N Navarra, Jeffrey R Petrella, Andy J Liu, Salman S Ikramuddin, P. Murali Doraiswamy

**Affiliations:** 1 Psychiatry, Duke University School of Medicine, Durham, USA; 2 Radiology, Duke University School of Medicine, Durham, USA; 3 Neurology, Duke University School of Medicine, Durham, USA

**Keywords:** alzheimer’s disease, amyloid-related imaging abnormalities (aria), anti-amyloid-β monoclonal antibodies, aria, cerebral amyloid angiopathy, mild cognitive impairment (mci)

## Abstract

We report a case of probable cerebral amyloid angiopathy (CAA) presenting with almost 100 cerebral microhemorrhages in an apolipoprotein E 4 (APOE4) homozygous subject with mild cognitive impairment (MCI). CAA is highly prevalent in autopsy studies and may impact over 100 million individuals worldwide; however, it remains substantially underdiagnosed clinically. CAA raises the risk for intracerebral hemorrhages as well as amyloid-related imaging abnormalities (ARIA) with amyloid-targeted antibodies. CAA is especially a quandary in APOE4 homozygotes who are at greatest risk for both Alzheimer's disease (AD) and ARIA. This case highlights the urgent need for greater diagnostic awareness and therapeutic development.

## Introduction

Cerebral amyloid angiopathy (CAA), first described in 1909, remains a dilemma more than a 100 years later. At autopsy, CAA pathology is found in approximately 79% of those with Alzheimer's disease (AD) and about 29% of cognitively normal elderly [1]. Unlike AD, CAA is characterized primarily by the deposition of amyloid-β40 in the media and adventitia of small cerebral and meningeal vessels; this, in turn, increases the risk of intracerebral hemorrhages, siderosis, and infarcts leading to cognitive impairment [2,3].

CAA can be classified into three major types: sporadic, the most common and occurring spontaneously without a known genetic mutation; familial, due to a specific genetic mutation; and iatrogenic, due to a medical procedure or treatment. The revised Boston 2.0 criteria are used to diagnose CAA as definite, probable, and possible [4].

The main aim of this case is to highlight the therapeutic challenges posed by CAA in the era of amyloid-targeted antibodies.

## Case presentation

A 75-year-old male with a history of exercise-induced asthma, supraventricular tachycardia, sleep apnea, osteopenia, hyperlipidemia, prediabetes, basal cell carcinoma, chronic back pain, anxiety, depression, and insomnia presented with a 2-year history of concerns with memory, attention, word finding, and executive functions.

He had no history of antithrombotic medication use, no neurological symptoms (other than cognitive complaints), and no history of transient focal neurological episodes (TFNE). Known vascular risks include hypercholesterolemia, supraventricular tachycardia, and sleep apnea. He was on several medications, including atorvastatin, mirtazapine, lithium orotate, citicoline, creatine, and tamsulosin.

An MRI done six months earlier revealed mild prominence of the cisterna magna and mild generalized parenchymal atrophy. It noted scattered foci of old microhemorrhages intracranially. There were no acute hemorrhages or infarcts. Mild patchy T2/FLAIR (fluid-attenuated inversion recovery) hyperintensity in the periventricular white matter with scattered foci and small patchy areas of similar white matter signal abnormality suggestive of chronic small vessel disease.

His grandmother experienced severe memory loss in her 50s, his mother was diagnosed with Alzheimer’s in her mid-70s, and his father was diagnosed with Alzheimer’s in his late 80s. He is homozygous for apolipoprotein E 4 (APOE4) and reports difficulty recalling names, a declining ability to play games and repeat stories, and misplacing objects. His Montreal Cognitive Assessment (MoCA) scores were 26 and 24 seven months later, with impaired delayed recall on testing [5]. His cerebral spinal fluid (CSF) p-Tau/Abeta42 ratio was abnormally elevated at 0.098.

**Figure 1:**
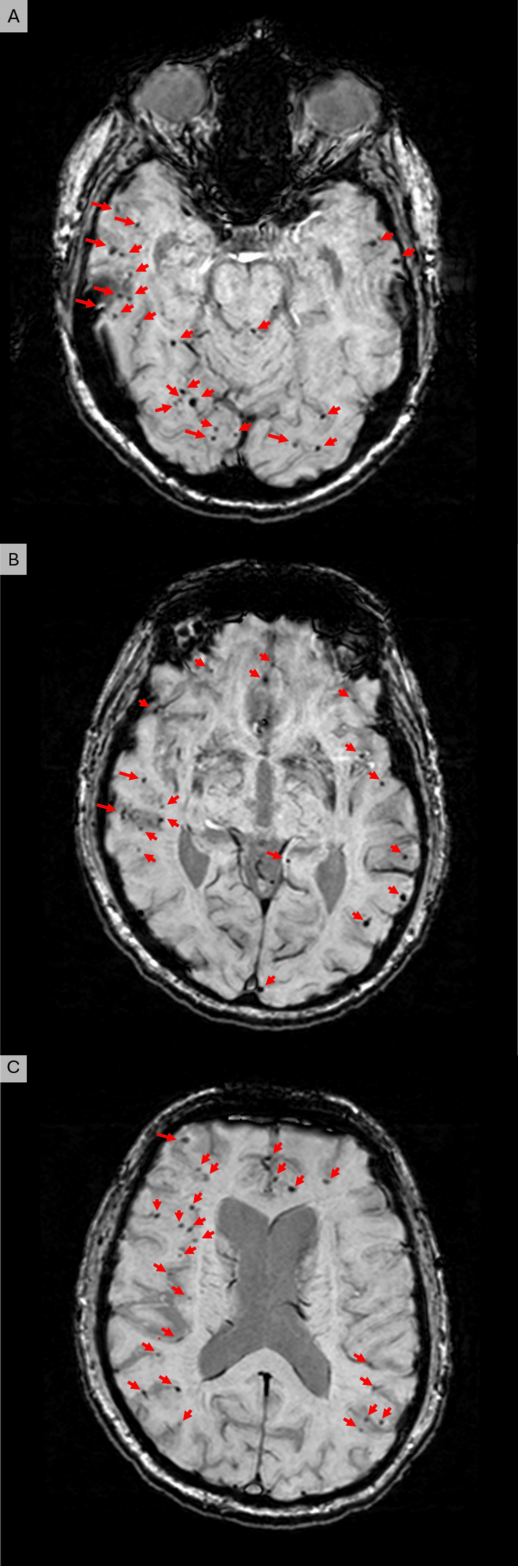
MR images obtained with a susceptibility-weighted imaging sequence Images obtained from the level of the midbrain, inferiorly (A) through the basal ganglia (B), to the corona radiata superiorly (C). The images demonstrate innumerable microhemorrhages (red arrows) throughout both cerebral hemispheres in a predominantly lobar distribution, consistent with the diagnosis of cerebral amyloid angiopathy. Red arrows indicate microhemorrhages.

His brain MR scan showed age-appropriate atrophy, and susceptibility weighted imaging (SWI) showed innumerable (approximately 100) cortical and subcortical microhemorrhages in a lobar distribution (Figure 1, red arrows). The distribution involves the entire supratentorial brain with clustering in the bilateral temporal, parietal, and occipital lobes. There were no additional findings, such as superficial siderosis, prominent perivascular spaces, or a multi-spot white matter pattern. There were no demonstrable deep gray or white matter or infratentorial microhemorrhages to suggest superimposed hypertensive microhemorrhage. He had no history of intracranial hemorrhages.

He was diagnosed with probable CAA as well as mild cognitive impairment (MCI) due to AD neuropathological change. He was started on donepezil 5 mg daily for 6 weeks, which was then increased to 10 mg daily, and he was advised to control his blood pressure and low-density lipoprotein levels. He was also advised to avoid anticoagulants and antiplatelet agents (unless there was a strong indication) and to continue exercise, lifestyle interventions, and staying social. His future risk for intracranial hemorrhage is lower than that of APOE2 carriers but remains high due to the extensive vascular involvement. Because of his genetic and clinical status, his prognosis was likely further progression of CAA and cognition. In addition to taking donepezil and following up with his neurologist, he opted to enroll in a 78-week trial of computerized cognitive training through which he will also receive periodic neuropsychological and daily activities assessments, plus a follow-up MRI scan, as well as cognitive remediation. He has remained clinically stable at our last interaction.

## Discussion

Two amyloid-targeted antibodies (ATTs), lecanemab and donanemab, are in routine clinical use for treating MCI and mild AD in several countries [6]. An important adverse effect of these agents is amyloid-related imaging abnormalities (ARIA) in the form of edema, superficial siderosis, or microhemorrhages, which can sometimes be serious or even fatal [6]. People who are APOE4 carry the highest risk (up to three times more) for developing ARIA, including serious forms, while on ATTs [6]. The presence of more than four microhemorrhages or superficial siderosis on a baseline MR scan is an exclusion criterion to initiate ATTs in the appropriate use criteria [7]. However, more recently, the drug labels have recognized that even the presence of two microhemorrhages can raise the risk for ARIA. MCI, especially in APOE4, is a critical stage for early intervention to preserve function. However, our subject was advised against ATTs, given the heightened risk for both ARIA and serious macrohemorrhages.

The relatively large number of microbleeds noted in our subject is unusual, as the majority of CAA subjects present with less than 16 microbleeds [8]. APOE4 increases the risk for CAA and amyloid deposition [2-16], which, along with hypercholesterolemia in this subject, may have led to the vast number of microhemorrhages seen. In a recent clinical trial of valiltramiprosate in APOE4/4 with MCI or mild AD [9], less than 1% had more than 100 microhemorrhages, indicating that such cases are rare. Transgenic AD mice models expressing human APOE4 have prominent CAA, and in such models, an anti-human APOE antibody was able to reduce CAA and improve cerebrovascular function [14]. The mechanisms by which APOE4/4 promotes CAA (and ARIA) are not fully understood but may involve the activation of the microglial Fc-gamma receptor [14].

The clinical manifestations of cerebral microbleeds are variable, but there is strong evidence that higher numbers of microbleeds are associated with greater severity of cognitive deficits as well as depression and emotional lability [8]. The location is also crucial; for example, frontal lobe microbleeds could be expected to impact working memory, attention, and psychomotor speed [8,10,11]. In this patient, the lobar distribution of microhemorrhages, likely involving the association cortex, could potentially contribute to subtle deficits in multiple domains, including executive function, visuospatial processing, and attention/processing speed, as well as receptive language impairment, given the involvement of posterior temporal language regions. Such deficits could add to the burden of an underlying AD process mediated by the APOE4 genotype and cortical amyloid deposition.

Prevalence estimates from autopsy and observational studies suggest that between 8%-25% of the elderly without dementia may have CAA [1-3,15,16]. However, in routine clinical practice, CAA remains substantially underdiagnosed, with prevalence rates less than 50% of those found at autopsy [1], primarily due to variability in applying diagnostic criteria, use of insensitive MR sequences, and/or lack of attributable symptoms. The implications of such CAA underdiagnosis on ARIA risk, as well as on cognitive outcomes in patients receiving ATTs, need further study [1,3,6,11]. A recent study examined the comparative sensitivity of 7T versus routine (1.5 or 3T) MRI for the detection of CAA markers in 40 subjects who underwent both scans and T2* and SWI sequences [12]. The detection rate of microbleeds and superficial siderosis on 7T T2* was two to three times greater than with conventional MR, highlighting the underdiagnosis gap [12].

Currently, there are no approved disease-modifying therapies for CAA patients beyond control of blood pressure and avoidance of anticoagulants. The unmet needs are particularly great in APOE4 or those with a large number of baseline microhemorrhages, who are also at the highest risk for ARIA. Investigational therapies being considered for CAA include mivelsiran (RNAi against amyloid precursor protein), vasoactive agents (cilostazol, taxifolin), minocycline (inhibitor of perivascular matrix degradation), valiltramiprosate, APOE antibodies, and anti-inflammatory agents [13].

## Conclusions

Given that there are more than a billion people over the age of 60, a projection of the 8-25% prevalence in the elderly population suggests that CAA impacts about 100 million people worldwide at the lower range of the estimate and about 250 million people at the higher end. These estimates highlight the large underdiagnosis gap.

Our case also supports prior research that has highlighted the importance of using sensitive MR sequences to increase CAA identification for both clinical care, such as consideration of ATTs, and research studies. Further research on developing early diagnostic markers and new therapies for CAA, as well as studies examining the links between CAA and ARIA, should be a priority. Our case highlights the unmet therapeutic needs of such individuals.
